# Rethinking the Accessibility of Hearing Assessments for Children with Developmental Disabilities

**DOI:** 10.1007/s10803-024-06461-9

**Published:** 2024-07-18

**Authors:** Angela Yarnell Bonino, Deborah Mood, Mary S. Dietrich

**Affiliations:** 1 Department of Hearing and Speech Sciences and Vanderbilt Kennedy Center, Vanderbilt University Medical Center, 1215 21st Ave South, Medical Center East - South Tower, Nashville, TN 37232 USA; 2 Department of Pediatrics, Section of Neurodevelopmental Behavioral Pediatrics, University of Colorado School of Medicine, Aurora, CO USA; 3 Department of Biostatistics, School of Nursing, Vanderbilt University, Nashville, TN USA

**Keywords:** Audiology, Autism, Down syndrome, Cerebral palsy, Intellectual disability, Audiogram, Auditory brainstem response, Health disparities, Pediatrics

## Abstract

We aim to determine the accessibility of gold-standard hearing assessments – audiogram or auditory brainstem response (ABR) – during the first 3 months of hearing health care for children with and without developmental disabilities. Electronic health records were examined from children (0–18 years) who received hearing health care at three hospitals. Children with developmental disabilities had a diagnosis of autism, cerebral palsy, Down syndrome, or intellectual disability. Assessments from the first 3 months were reviewed to determine if ≥ 1 audiogram or ABR threshold was recorded. To evaluate differences in assessment based on disability status, logistic regression models were built while accounting for age, race, ethnicity, sex, and site. Of the 131,783 children, 9.8% had developmental disabilities. Whereas 9.3% of children in the comparison group did not access a gold-standard assessment, this rate was 24.4% for children with developmental disabilities (relative risk (RR) = 3.79; *p* < 0.001). All subgroups were at higher risk relative to the comparison group (all *p* < 0.001): multiple diagnoses (RR = 13.24), intellectual disabilities (RR = 11.52), cerebral palsy (RR = 9.87), Down syndrome (RR = 6.14), and autism (RR = 2.88). Children with developmental disabilities are at high risk for suboptimal hearing evaluations that lack a gold-standard assessment. Failure to access a gold-standard assessment results in children being at risk for late or missed diagnosis for reduced hearing. Results highlight the need for (1) close monitoring of hearing by healthcare providers, and (2) advancements in testing methods and guidelines.

## Introduction

Determining a child’s hearing status in a timely manner is important for an accurate differential diagnosis and to access appropriate hearing-related interventions for maximizing developmental outcomes (Cupples et al., 2018; Moeller, 2000; Yoshinaga-Itano et al., 1998, 2017). For infants, the early hearing detection and intervention framework is 1-3-6: screen by 1 month, diagnosis by 3 months, and start intervention by 6 months of age (JCIH 2019). In contrast, despite the common onset of reduced hearing after the neonatal period (Dumanch et al., 2017; Su & Chan, 2017), there are not well-established criteria for what is considered a timely diagnosis or access to hearing technology at older ages. What is clear is that long delays often occur despite substantial evidence that early diagnosis and access to interventions leads to better outcomes across the lifespan (Meinzen-Derr et al., 2020; Powell et al., 2021; Stiles et al., 2020; Walker et al., 2014; Yoshinaga-Itano, 2003). Thus, when hearing concerns are raised at any age, timely diagnosis is essential.

Pediatric audiology practice guidelines recommend a test battery approach using behavioral and physiological measures (AAA 2020; ASHA 2004). At the core of the battery are two gold-standard measures: the audiogram and auditory brainstem response (ABR). Using behavioral methods, the audiogram is the lowest intensity level (i.e., threshold) frequency-specific signals can be detected. The audiogram is the most reliable and accurate measure of hearing and can be obtained starting around 6 months of age in infants with typical development (Gravel & Traquina, 1992). When behavioral testing cannot be successfully performed, hearing sensitivity is evaluated with ABR. ABR is a physiological measure of auditory pathway integrity from the cochlea to the brainstem. During ABR testing, a child needs to be quiet and still. To achieve this requirement, testing is performed under natural sleep prior to 6 months of age, and sedation thereafter. Audiogram or ABR thresholds, are then cross-checked with speech audiometry and physiological measures (i.e., tympanometry, otoacoustic emissions (OAEs), or middle ear muscle reflexes). Completing a gold-standard assessment is an important benchmark in hearing health care because audiogram or ABR thresholds are required to prescribe hearing technology and determine eligibility for hearing-related early intervention or special education services.

One population that experiences barriers to timely and high-quality hearing health care are children with developmental disabilities. Developmental disabilities are a group of conditions that result in early onset of impairments in motor, cognitive, communication, sensory, social, and/or emotional functioning that are expected to persist throughout the lifespan. Moreover, many developmental disabilities commonly co-occur with reduced hearing. Relative to the general population, the prevalence of reduced hearing is well established to be higher for children with cerebral palsy (CP), Down syndrome (DS), or intellectual disability (ID), whereas results are mixed for children with autism spectrum disorder (ASD) (De Schrijver et al., 2019; Erickson & Quick, 2017; Hild et al., 2008; Kreicher et al., 2018; Meuwese-Jongejeugd et al., 2006; Mishaal et al., 2022; Reid et al., 2011; Rosenhall et al., 1999; Shott et al., 2001; Weir et al., 2018; Willems et al., 2022). Additionally, converging evidence indicates that children with developmental disabilities – including those with ASD, CP, DS, and ID – experience long referral delays for ABR testing, high rates of missed or late identification of reduced hearing, and low use rates for hearing technology (Basonbul et al., 2020; Haller et al., 2022; Hild et al., 2008; Meinzen-Derr et al., 2014; Nightengale et al., 2020; Richard et al., 2021; Trudeau et al., 2021). Failure to meet the same assessment standards or to achieve timely diagnosis and intervention for this population reflects an inequity in care that compounds the impact of reduced hearing on developmental outcomes (Cupples et al., 2018; Meinzen-Derr et al., 2011; Yoshinaga-Itano et al., 2017).

Although the underlying factors for these disparities are likely multifaceted, accessibility is a common barrier experienced by persons with disabilities (e.g., lack of accessible medical facilities, examination equipment, or individualized accommodations). In the context of hearing assessments, accessibility barriers can be the result of testing requirements and methods that are misaligned with the unique developmental or medical needs of children with developmental disabilities. For example, behavioral testing methods are grounded in the assumptions of typical child development across multiple domains. Thus, audiologists often have difficulty obtaining an audiogram from children with developmental disabilities (Gans & Gans, 1993; Greenberg et al., 1978; Meagher et al., 2021; Nightengale et al., 2020; Tharpe et al., 2006; Trudeau et al., 2021). For ABR testing, concomitant health problems in this population may contraindicate the use of sedation (Basonbul et al., 2020; Trudeau et al., 2021).

The purpose of the present study was to determine the accessibility of gold-standard hearing assessments based on a diagnosis of ASD, CP, DS, or ID across multiple sites. Here we define accessibility as both the availability of a gold-standard assessment and the ability to successfully use it to collect required information to determine hearing status. Thus, we determined if at least one audiogram and/or ABR threshold was recorded in the electronic health record within 3 months of the child’s initial encounter in the audiology clinic. The rationale for a 3-month period is that this allows for the possibility of multiple encounters to complete a comprehensive hearing evaluation, yet is an acceptable duration based on the benchmarks for early identification of reduced hearing (ASHA 2004; JCIH 2019). To our knowledge, this is the first study to characterize the current state of assessment practices for both behavioral audiometry and ABR by harnessing electronic health records from multiple sites. Although using electronic health records introduces a variety of potential errors (Bastarache, 2021), this approach is a critical next step in establishing inclusive pediatric hearing health care by determining the accessibility of gold-standard assessments – the gateway to hearing technology, visual communication, and early intervention or school-based services. Moreover, this work is aligned with the national health priority set by *Health People 2030* to improve health and well-being in persons with disabilities in the United States (Office of Disease Prevention and Health Promotion, n.d.).

## Materials and Methods

Electronic health records were accessed from 162,554 children (0–18 years) through the Audiological and Genetic Database (AudGenDB) (Pennington et al., 2020). AudGenDB is a HIPAA-compliant, publicly available, online database containing de-identified audiological and medical health record data. Children received hearing health care at three academic hospitals in the United States: Children’s Hospital of Philadelphia, Vanderbilt University Medical Center, or Boston Children’s Hospital. All encounters occurred between 2008 and 2018, but the duration of the data capture period differed across sites. This study was deemed exempt by the Institutional Review Board.

We extracted and used all available pediatric patients from the registry who had (1) any type of hearing assessment data in their record, and (2) one or more International Classification of Diseases 9th or 10th revision (ICD-9 or ICD-10) diagnosis codes in their record. Allowable assessment types were audiogram, ABR, speech audiometry, tympanometry, OAEs, and middle ear muscle reflex testing. Encounters with audiogram or ABR data were required to have at least one threshold obtained with any stimulus or transducer type. Speech audiometry encounters had a detection or recognition threshold, or a word recognition score. The last three measures are physiologic measures: tympanometry to evaluate middle ear status; OAEs to measure outer hair cell functioning in the cochlea; and middle ear muscle reflexes to check the integrity of the neural pathways surrounding the stapedial reflex. These measures were required to have a recorded value; “absent” or “no response” were permitted. For all measures, data were only required for a single ear. Non-ear specific data were permitted for audiogram and speech audiometry encounters. We excluded audiogram or speech audiometry testing conducted with hearing technology. Any ICD codes captured by the registry were permitted to meet inclusion criteria for the study. We only included data that were recorded prior to 19 years of age.

Because billing code practices often result in hearing-related ICD codes being unreliable, hearing status was determined based on the first audiogram or ABR encounter with sufficient information to allow classification. A child was identified with reduced hearing if the pure-tone average (PTA) was > 25 dB HL on the audiogram. A PTA-4 was computed by averaging 0.5, 1.0, 2.0, and 4.0 kHz, but a PTA-3 (0.5, 1.0, 2.0 kHz) was used if data were not available at 4.0 kHz. The PTA was computed from thresholds collected at the same encounter, but separate encounters were permitted between ears. Soundfield thresholds were used if ear-specific results were unavailable. This method was selected because previous work by Bonino and Mood (2023) indicated that this classification method resulted in high stability of hearing status classification over time and showed high rates of inclusion of children with developmental disabilities. For ABR, children were identified with reduced hearing if the across-threshold average (minimum of 2; 0.5, 1.0, 2.0, or 4.0 kHz) or a click stimulus was > 30 dB nHL (Ontario Infant Hearing Program, 2020). If a child had a hearing status classification from both audiogram and ABR data, the earlier results were used. Hearing status was classified as “unknown” if there were no or too few thresholds in the record to determine hearing status based on the above methods.

Children were classified based on their disability status, sex, race, and ethnicity. Using the ICD-9 and ICD-10 diagnostic codes, (Supplemental Table 1), we identified children with a diagnosis of ASD, CP, DS, or ID in their record. Children with one or more of these diagnoses were assigned to the developmental disability group. Children who did not have any of the required codes were assigned to the comparison group. Race was categorized into 5 groups: White, Black or African American, Asian, Indigenous (Indian, Native Alaskan, American Indian, Native Hawaiian, and Pacific Islander), and Other/Multiple Race. Ethnicity was categorized as Hispanic/Latino or non-Hispanic/Latino.

### Statistical Procedures

We used data cleaning techniques in *Python* (version 3.0) to manipulate the multiple comma-separated value (CSV) files to extract demographics, encounter history, and clinical data. *IBM SPSS Statistics* (version 28.0) was used to generate descriptive summaries of variables by site and to inform appropriate analytic approaches. Binomial (binary categorical dependent variables) and multinomial (≥ 2 categorical outcome variables) logistic regressions were conducted using *Stata* (version 15) for our primary analyses. Regression models included the child’s age at first encounter, sex, race, and ethnicity as covariates and accounted for nesting within each of the 3 sites by using the “vce(cluster)” option in *Stata*. Children with missing race or ethnicity data were coded as “other” in these models. We generated odds ratios (OR) and Cohen’s *d* effect statistics for the unadjusted, descriptive comparisons of the groups separated by disability status. Relative risk (RR) ratios with 95% confidence intervals were generated from regression models. We used an alpha of 0.05 (*p* < 0.05) to determine statistical significance.

## Results

### Overview of Patients

The final dataset included 131,783 children. The care site was Children’s Hospital of Philadelphia for 74.5% of the children, Boston Children’s Hospital for 14.6%, and Vanderbilt University Medical Center for 10.9%. A diagnosis of a developmental disability was identified for 12,867 children (9.8%): 8,166 had ASD (6.2%); 2,665 had DS (2.0%); 2,252 had ID (1.7%); and 2,095 had CP (1.6%). Of children with developmental disabilities, 1,988 (15.5%) had two or more diagnoses. As summarized in Table 1 and replicating prior work, the developmental disability group had a higher percentage of children who identified as boys, belonged to a racial minority group, and were Hispanic/Latino than did the comparison group (all *p* < 0.001) (McGuire et al., 2019; Zablotsky et al., 2019). The median age at first encounter was 3.2 years for children with developmental disabilities and 3.0 years for children in the comparison group. Both groups had a mean of one encounter in the initial 3-month period. No significant differences were observed between the two groups for these two variables. Given that the expected population differences in age at first encounter, sex, race, and ethnicity existed between the groups in this study, in addition to accounting for the clustering of observations within the three organizational sites, all analyses of group differences were adjusted for those known confounding characteristics.

**Table 1:** Characteristics of children in the final sample separated by developmental disability status

Developmental Disability Status						
	*N*	No *n* (%)	Yes *n* (%)	OR	95% CI	*p*-value
**Total**		118,916 (90.2)	12,867 (9.8)			
**Sex** ^**a**^						
Female ^d^	53,663	49,695 (92.6)	3,968 (7.4)			
Male	78,120	69,221 (88.6)	8,899 (11.4)	1.61	1.54,1.68	< 0.001
**Race** ^**b**^						
White ^d^	89,076	81,222 (91.2)	7,854 (8.8)			
Asian	4,372	3,771 (86.3)	601 (13.7)	1.65	1.50, 1.81	< 0.001
Black	19,836	17,333 (87.4)	2,503 (12.6)	1.49	1.42, 1.57	< 0.001
Indigenous	196	169 (86.2)	27 (13.8)	1.65	1.10, 2.49	0.016
Other/ Multiple	18,303	16,421 (89.7)	1,882 (10.3)	1.19	1.12, 1.25	< 0.001
**Hispanic or Latino** ^**c**^						
No ^d^	95,280	85,115 (89.3)	10,165 (10.7)			
Yes	10,079	8,756 (86.9)	1,323 (13.1)	1.27	1.19, 1.35	< 0.001
		**Median (IQR)** **Min**,** Max**	**Median (IQR)** **Min**,** Max**			
**Age at First Encounter** **(Years)**		3.2 (1.5, 6.5) < 1 day, 19 years	3.0 (1.7, 6.4) < 1 day, 19 years	1.00	0.99, 1.01	0.134 ^e^
**Number of Encounters (Initial 3 Months)**		1 (1, 2) 1, 15	1 (1, 2) 1, 10	1.02	0.99, 1.05	0.148 ^f^

^a^ Missing = 15 (< 1%); ^b^ Missing = 7,378 (6%); ^c^ Missing = 26,424 (20%); imputed data summaries displayed

^d^ Referent category for logistic regression

^e^ Cohen’s *d* = 0.02; ^f^ Cohen’s *d* = 0.001

OR = Odds ratio

CI = Confidence interval

Hearing status by developmental disability diagnoses are reported in Table 2. Relative to the comparison group, children with developmental disabilities were significantly more likely to be classified as having unknown hearing status (RR = 1.24; CI = 1.20, 1.28; *p* < 0.001). All disability subgroups had a higher risk for unknown hearing status (*p* < 0.05) except for children with Down syndrome (*p* = 0.091). Two disabilities were associated with a higher risk of reduced hearing than seen in the comparison group (18.7%): CP (23.8%; RR = 2.31; CI = 1.02, 5.24; *p* = 0.044) and DS (31.9%; RR = 2.17; CI = 1.01, 4.64; *p* = 0.047).

**Table 2:** Summaries of hearing status classification as determined by the first available encounter with sufficient audiogram or ABR thresholds^a^

		Hearing Status Classification		
	*N*	Typical *n* (%)	Reduced *n* (%, *p*-value) RR (95% CI)	Unknown ^b^ *n* (%, *p*-value) RR (95% CI)
**Total**	131,783	85,664 (65.0)	24,570 (18.6)	21,549 (16.4%)
**Developmental Disability Status**				
None	118,916	78,794 (66.3)	22,231 (18.7)	17,891 (15.0)
Any Diagnosis	12,867	6,870 (53.4)	2,339 (18.2, 0.281) 1.47 (0.72. 2.96)	3,658 (28.4, < 0.001) **1.24** (1.20, 1.28)
1 Diagnosis	10,879	6,078 (55.9)	1,838 (16.9, 0.297) 1.35 (0.76, 2.38)	2,963 (27.2, < 0.001) **1.23** (1.19, 1.28)
2 or More Diagnoses	1,988	792 (39.8)	501 (25.2, 0.264) 2.03 (0.58, 7.05)	695 (35.0, 0.027) **1.27** (1.02, 1.58)
**Specific Disability** ^**c**^				
Autism	8,166	4,940 (60.5)	1,014 (12.4, 0.622) 0.86 (0.48, 1.55)	2,212 (27.1, 0.022) **1.36** (1.04, 1.78)
Cerebral Palsy	2,095	779 (37.2)	498 (23.8, 0.044) **2.31** (1.02, 5.24)	818 (39.0, 0.022) **1.84** (1.09, 3.12)
Down Syndrome	2,665	1,088 (40.8)	849 (31.9, 0.047) **2.17** (1.01, 4.64)	728 (27.3, 0.091) 0.79 (0.59, 1.04)
Intellectual Disability	2,252	955 (42.4)	589 (26.2, 0.470) 1.67 (0.41, 6.76)	708 (31.4, 0.032) **1.49** (1.03, 2.15)

^a^ Bolded RR values indicate a statistically significant difference relative to the comparison group (no diagnosis)

^b^ Unknown hearing status was defined as insufficient threshold data in the cumulative record

^c^ May have more than one disability

RR = Relative risk adjusted for site, age 1st encounter, sex, race, Hispanic/Latino

CI = Confidence interval

### Access to Gold-Standard Diagnostic Assessments

To determine if children had access to an audiogram and/or an ABR test, we examined the initial encounters and all subsequent encounters for the next 3 months. As shown in Table 3, an audiogram was accessed by 89.2% of children in the comparison group and 72.8% of children in the developmental disability group. ABR was infrequently accessed (1.5% of the total sample), but children with developmental disabilities were more likely to access ABR testing than those in the comparison group (RR = 3.72; CI = 2.16, 6.38; *p* < 0.001).

**Table 3:** Summaries of the percentage of children who accessed audiogram testing, ABR testing, both (audiogram and ABR), or neither (no threshold for either assessment type) during the initial 3 months of hearing health care

	*N*	Neither (*N* = 14,140)	Audiogram ^a^ (*N* = 115,422)	ABR (*N* = 2,031)	Both (*N* = 190)
		% (*p*-value) RR (95% CI)	%	% (*p*-value) RR (95% CI)	% (*p*-value) RR (95% CI)
**Total**	131,783	10.7%	87.6%	1.5%	0.1%
**Developmental Disability Status**					
None	118,916	9.3%	89.2%	1.4%	0.1%
Any Diagnosis	12,867	24.4% (< 0.001) 3.79 (3.08, 4.67)	72.8%	2.5% (< 0.001) 3.72 (2.16, 6.38)	0.3% (0.003) 3.86 (1.56, 9.56)
1 Diagnosis	10,879	22.8% (< 0.001) 3.16 (2.66, 3.75)	74.9%	2.1% (< 0.001) 2.63 (1.65, 4.18)	0.3% (0.030) 3.28 (1.12, 9.54)
2 or More Diagnoses	1,988	33.2% (< 0.001) 13.24 (6.26, 27.99)	61.4%	5.0% (< 0.001) 25.69 (5.78, 114.20)	0.4% (< 0.001) 12.48 (5.27, 29.52)
**Specific Disability** ^**b**^					
Autism	8,166	20.5% (< 0.001) 2.88 (1.75, 4.73)	77.7%	1.5% (< 0.001) 4.04 (1.96, 8.31)	0.3% (0.005) 2.84 (1.36, 5.92)
Cerebral Palsy	2,095	37.8% (< 0.001) 9.87 (8.76, 11.13)	55.5%	6.0% (< 0.001) 13.55 (5.89, 31.13)	0.7% (< 0.001) 14.62 (5.06, 42.22)
Down Syndrome	2,665	30.6% (< 0.001) 6.14 (5.01, 7.53)	65.3%	3.9% (0.018) 3.72 (1.25, 11.04)	0.2% (0.127) 2.77 (0.74, 10.25)
Intellectual Disability	2,252	28.1% (< 0.001) 11.52 (5.52, 24.04)	67.1%	4.3% (< 0.001) 29.18 (6.39, 133.10)	0.5% (< 0.001) 14.36 (7.82, 26.36)

^a^ Referent outcome category

^b^ May have more than one disability

RR = Relative risk adjusted for site, age 1st encounter, sex, race, Hispanic/Latino

CI = Confidence interval

For the comparison group, 9.3% of children did not access either of the gold-standard assessments. In contrast, 24.4% of children with at least one diagnosed developmental disability did not access either gold-standard assessment (RR = 3.79; CI = 3.08, 4.67; *p* < 0.001). Moreover, the risk of no access was significantly higher for children with two or more diagnoses (33.2%; RR = 13.24; CI = 6.26, 27.99; *p* < 0.001). Relative to the comparison group, all disability subgroups were significantly more likely to not access a gold-standard assessment in the 3-month period (all *p* < 0.001) and varied from 20.5% for the group with ASD (RR = 2.88; CI = 1.75, 4.73) to 37.8% for those diagnosed with CP (RR = 9.87; CI = 8.76, 11.13). Results from a supplemental analysis were not consistent with scheduling delays being the reason for the lack of access (Supplemental Table 2). Specifically, only 19.8% of children in the sample who did not access audiogram or ABR testing at 3 months had accessed it by 12 months.

A supplemental analysis on testing access was conducted to explore the contributions of co-occurring ID for children with ASD or CP. As shown in Supplemental Table 3, children with a sole diagnosis of ASD were more likely to access a gold-standard assessment than children with a dual diagnosis of ASD and ID (81.3% vs. 74.5%; *p* < 0.001). The same pattern was seen for children with CP (66.3% vs. 47.8%; *p* < 0.001). Thus, co-occurring ID further increases the risk of children with ASD or CP for not accessing a gold-standard assessment during the initial 3-month period. These results should be interpreted cautiously because ID may be underreported in this dataset.

### Diagnostic Care for Children Who Did Not Access A Gold-Standard Assessment

The course of diagnostic care for children who did not access a gold-standard hearing assessment warranted further exploration. Table 4 summarizes the patterns of use for the secondary hearing assessment measures during the initial 3-month period. Tympanometry was nearly universally accessed (94.1%). For the remaining measures, there were missing data across all sites and results should be interpreted cautiously. Across the available sites, 18.3% of children accessed OAEs, 32.4% accessed speech audiometry, and 11.9% of children accessed middle ear muscle reflex testing.

**Table 4:** The usage of secondary hearing assessments by site and developmental disability status during the first 3 months of hearing health care for the children who lacked a gold-standard assessment during that initial period (*N* = 14,140)

		Site		
Secondary Assessment Type	Total (*N* = 14,140) *n* (%)	BCH (*N* = 4,706) *n* (%)	CHOP (*N* = 6,050) *n* (%)	VUMC (*N* = 3,384) *n* (%)
**Tympanometry**	13,300 (94.1)	4,705 (100.0)	5,554 (91.8)	3,041 (89.9)
No DD	93.6%	100.0%	90.7%	89.9%
DD	95.7%	100.0%	95.2%	89.7%
**Otoacoustic Emissions**	1,725 (18.3) ^a^	n/a	1,620 (26.8)	105 (3.1)
No DD	17.8%	n/a	27.3%	1.8%
DD	19.8%	n/a	25.0%	8.7%
**Speech Audiometry**	3,052 (32.4) ^a^	n/a	858 (14.2)	2,194 (64.8)
No DD	35.0%	n/a	14.1%	70.4%
DD	23.1%	n/a	14.3%	41.9%
**Middle Ear Muscle Reflexes**	962 (11.9) ^b^	137 (2.9)	n/a	825 (24.4)
No DD	11.5%	3.1%	n/a	22.9%
DD	13.3%	2.4%	n/a	30.5%

Note: “n/a” the site did not administer the specific test

^a^ Total *N* = 9,434

^b^ Total *N* = 8,090

DD = Developmental disability

BCH = Boston Children’s Hospital

CHOP = Children’s Hospital of Philadelphia

VUMC = Vanderbilt University Medical Center

Table 5 reports the prevalence of reduced hearing for the subset of children (*N* = 3,999) who did not access a gold-standard assessment in the initial 3 months but ultimately had sufficient thresholds to determine hearing status because they accessed testing later. Overall, 28.7% of these children were subsequently classified as having reduced hearing. Although the percentage of children classified with reduced hearing was higher for the developmental disability group than the comparison group (37.4% vs. 26.5%), this difference was not statistically significant (*p* = 0.138). However, there was considerable variability across the disability subgroups. Children with ASD had a similar rate of reduced hearing as children in the comparison group (27.5%; *p* = 0.803). To the contrary, the likelihood of reduced hearing was significantly higher for children diagnosed with CP (50.6%; RR = 2.63; CI = 1.36, 5.08; *p* = 0.004) or DS (45.8%; RR = 2.31; CI = 1.14, 4.68; *p* = 0.020) relative to the comparison group. A high number of children with ID were classified with reduced hearing, but their risk estimate was not significantly different from the comparison group (47.1%, RR = 1.98; CI = 0.69, 5.66; *p* = 0.205).

**Table 5:** Hearing status classification for a subset of children who did not access a gold-standard hearing assessment during the initial 3 months, but subsequently accessed audiogram or ABR testing and had sufficient threshold data to determine their hearing status ^a^

	*N*	Typical Hearing *n* (%)	Reduced Hearing *n* (%)	RR (95% CI)	*p*-value
**Total**	3,999	2,853 (71.3)	1,146 (28.7)		
**Developmental Disability Status**					
None	3,202	2,345 (73.5)	848 (26.5)		
Any Diagnosis	797	499 (62.6)	298 (37.4)	1.57 (0.86, 2.86)	0.138
1 Diagnosis	629	413 (65.7)	216 (34.3)	1.42 (0.86, 2.33)	0.168
2 or More Diagnoses	168	86 (51.2)	82 (48.8)	2.31 (0.88, 6.02)	0.086
**Specific Disability** ^**b**^					
Autism	346	251 (72.5)	95 (27.5)	0.94 (0.57, 1.54)	0.803
Cerebral Palsy	180	89 (49.4)	91 (50.6)	**2.63** (1.36, 5.08)	0.004
Down Syndrome	332	180 (54.2)	152 (45.8)	**2.31** (1.14, 4.68)	0.020
Intellectual Disability	140	74 (52.9)	66 (47.1)	1.98 (0.69, 5.66)	0.205

^a^ Bolded RR values are statistically significant

^b^ May have more than one disability

RR = Relative risk adjusted for site, age 1st encounter, sex, race, Hispanic/Latino

CI = Confidence interval

## Discussion

In this retrospective study of the initial 3-months of hearing health care, we found that children with developmental disabilities were nearly 4 times more likely not to access a gold-standard assessment than children in the comparison group. The highest relative risk (13 times) was for children with multiple diagnoses. Across diagnoses, the risk of not accessing a gold-standard assessment varied from a 3-fold increase for ASD to an 11-fold increase for ID. Consistent with previous literature indicating a high co-occurrence of reduced hearing and CP, DS, or ID (De Schrijver et al., 2019; Erickson & Quick, 2017; Hild et al., 2008; Kreicher et al., 2018; Meuwese-Jongejeugd et al., 2006; Reid et al., 2011; Shott et al., 2001; Weir et al., 2018), our results confirmed that children with DS or CP had a higher rate of reduced hearing than seen for the comparison group within this high-risk clinical population. These results confirm our hypothesis that children with developmental disabilities experience disparities in assessment despite being at high risk for reduced hearing. Consequently, children with developmental disabilities are at risk for missed or late diagnosis of reduced hearing which can have adverse consequences on development.

Our findings support the growing body of evidence that indicates that children with developmental disabilities have difficulty accessing hearing assessments. Previous laboratory studies using clinical behavioral methods have revealed that relative to typically developing, age-matched peers, fewer thresholds were obtained for infants and children with multiple disabilities (Gans & Gans, 1993); children with ASD (Tharpe et al., 2006); or infants with DS (Greenberg et al., 1978). Similarly, retrospective clinical studies have confirmed difficulty in collecting behavioral thresholds from children with ASD or DS (Basonbul et al., 2020; Meagher et al., 2021; Nightengale et al., 2020). Moreover, long ABR referral wait times are seen for children with developmental disabilities. The average period between first unsuccessful behavioral testing attempt and ABR testing was 9, 20, and 22 months for children with ASD, CP, and DS, respectively (Trudeau et al., 2021). In the present study, children with developmental disabilities were more likely to be tested with ABR than seen in the comparison group. However, given the high number of children with no gold-standard assessment, the access to ABR was inadequate to compensate for accessibility barriers for behavioral testing. A unique contribution of the present study is quantifying the relative risk across four disabilities (ASD, CP, DS, or ID) for not accessing a gold-standard hearing assessment during the initial period of hearing health care at three hospitals.

There is likely a multifaceted explanation for why we observed disparities. We recognize that pediatric audiologists strive to provide quality care to all children and make management decisions at the individual level that are driven by best intent. However, by harnessing the power of big data analytics with electronic health records across multiple sites, results from the present study spotlight that children with developmental disabilities receive disparate care. We posit that a primary factor is because behavioral testing methods are misaligned with the developmental abilities of children who have complex or diverse developmental profiles. Specifically, behavioral testing methods used in the clinic assume developmental mastery of skills such as object permanence, cause and effect, visual attention, inhibition, the ability to shift and divide attention, and in some cases, fine motor and play skills. When any of these skills are impacted by the child’s developmental disability it can make the audiogram inaccessible (Greenberg et al., 1978). Delays or failure to refer for ABR testing can happen due to repeated efforts to obtain behavioral testing or because of sedation concerns due to underlying medical conditions (Basonbul et al., 2020; Trudeau et al., 2021). Another source for possible factors contributing to the observed disparities is the model of care used in audiology clinics. Clinical procedures and systems (e.g., protocols and guidelines, scheduling practices, and space configuration) were designed based on the needs of children with typical development not those with disabilities. Lastly, it is important to consider that factors related to social determinants of health, including racism and disability-based discrimination, result in inequities in care that are expected to further compound with these accessibility factors (Ames et al., 2023; Kingsbury et al., 2022; Schuh & Bush, 2022). Future research is needed to determine the contributions of these factors to identify effective strategies for improving care.

Findings from this study reveal that there is a critical need for advancements in clinical care and research to address the lack of accessibility of gold-standard hearing assessments. To overcome the shortcoming of assuming typical development by current behavioral testing methods, it is paramount to design behavioral methods that take into consideration features of diverse or complex developmental profiles (e.g., difficulty shifting attention, longer response window due to motor planning delays, sensory sensitivities). In the interim, an as-of-now underutilized strategy in most audiology clinics is incorporating evidence-based supports and strategies that promote the learning of a new task, reduce anxiety, promote transitions, and improve engagement with an activity (Hume et al., 2021). For example, visual supports (e.g., picture schedules) can be used to help prepare a child for the test session and reduce anxiety (McTee et al., 2019; Roberts et al., 2020). Offering efficacious behavioral methods suitable for children with developmental disabilities has the potential to facilitate timely identification of reduced hearing.

A second clinical implication of this work is that audiologists need robust assessment protocols and timetables based on a child’s developmental profile and medical needs. This guidance should clarify what assessment data to prioritize based on common hearing profiles seen for specific developmental disabilities. Timetables should define what is an acceptable waiting period between unsuccessful behavioral testing and referring for a sedated ABR. Guidance is also needed on how soon children with developmental disabilities should return to the clinic for behavioral testing when an audiogram is incomplete. Protocols and timetables should provide clear guidance for when abnormal middle ear functioning is suspected. This is because children with developmental disabilities often have reduced hearing because of structural or functional differences in their middle ear space and/or increased prevalence of otitis media that does not resolve (Rosenfeld et al., 2016).

Another opportunity to improve early diagnosis is to expand eligibility criteria for infants who should receive a natural sleep, diagnostic ABR by 3 months of age regardless of their newborn hearing screening results. This recommendation has been advocated for all infants with craniofacial abnormalities, Down syndrome, or other syndromes associated with reduced hearing because of the high rate of ultimately being diagnosed with permanent reduced hearing (Basonbul et al., 2020; Ellis et al., 2023; Horn et al., 2021). Although additional research is needed, this recommendation may be appropriate for infant with or at high risk for CP (Richard et al., 2021). For these populations, performing a diagnostic ABR evaluation during early infancy would allow early diagnosis and provide valuable baseline information that can be used by hearing surveillance programs.

A final implication of this study is that medical or allied health professionals working with children with developmental disabilities should closely monitor for timeliness and completeness of hearing evaluation results based on established recommendations (JCIH 2019). Collaboration and coordination across providers on a child’s care team may facilitate determining hearing status. Providers should encourage families to continue the process of obtaining hearing data as multiple visits may be needed.

There are several limitations for this study that need to be considered. Because this study used retrospective electronic health record data from multiple sites it is vulnerable to errors commonly seen in clinical data. It is possible that sites changed their testing protocol or handling of records over the extraction period. Testing protocols differ across clinical sites and can differ among audiologists at a site. Due to the construction of AudGenDB, we cannot determine if gold-standard assessments were attempted but no thresholds were collected. However, even if a test was attempted, it is not accessible if thresholds are not collected to use for their intended purpose of determining hearing status and eligibility for hearing-related interventions. There can be group membership errors due to billing code errors or omissions. We used a narrow definition of developmental disabilities which means that children with other developmental disabilities were included in the comparison group. External validity of this study is restricted to pediatric-focused, academic hospitals. Assessment practices might be influenced by scheduling constraints and by appointment type. Expertise is expected to affect assessment practices. That is, sites included in this study, have large teams of audiologists, established protocols, and routinely see children with complex and diverse developmental profiles and medical needs.

## Conclusion

Many developmental disabilities commonly co-occur with reduced hearing. However, results from the present study confirm that children with developmental disabilities are nearly 4 times more likely not to access a gold-standard assessment than children in the comparison group. Thus, our results add to the growing body of evidence that children with developmental disabilities receive disparate hearing health care. The observed disparities highlight the need for advancements in hearing assessment testing methods and the development of tailored hearing assessment guidelines for children with developmental disabilities.

## Data Availability

A publicly available database was used in this study. The database can be found at https://audgendb.github.io/.

## Abbreviations

AudGenDB: Audiological and Genetic Database
ABR: Auditory Brainstem Response
ASD: Autism spectrum disorder
CP: Cerebral palsy
DS: Down syndrome
ID: Intellectual disability
ICD: International Classification of Diseases
OAE: Otoacoustic Emission
PTA: Pure-tone average
